# Comparison of the fit of automatic milking system and test-day records with the use of lactation curves

**DOI:** 10.5713/ajas.19.0190

**Published:** 2019-07-01

**Authors:** B. Sitkowska, M. Kolenda, D. Piwczyński

**Affiliations:** 1 Department of Biotechnology and Animal Genetics, Faculty of Animal Breeding and Biology, UTP University of Science and Technology, Mazowiecka 28, 85-084 Bydgoszcz, Poland

**Keywords:** Automatic Milking System, Lactation Curve, Test-day Milking, Cattle

## Abstract

**Objective:**

The aim of the paper was to compare the fit of data derived from daily automatic milking systems (AMS) and monthly test-day records with the use of lactation curves; data was analysed separately for primiparas and multiparas.

**Methods:**

The study was carried out on three Polish Holstein-Friesians (PHF) dairy herds. The farms were equipped with an automatic milking system which provided information on milking performance throughout lactation. Once a month cows were also subjected to test-day milkings (method A4). Most studies described in the literature are based on test-day data; therefore, we aimed to compare models based on both test-day and AMS data to determine which mathematical model (Wood or Wilmink) would be the better fit.

**Results:**

Results show that lactation curves constructed from data derived from the AMS were better adjusted to the actual milk yield (MY) data regardless of the lactation number and model. Also, we found that the Wilmink model may be a better fit for modelling the lactation curve of PHF cows milked by an AMS as it had the lowest values of Akaike information criterion, Bayesian information criterion, mean square error, the highest coefficient of determination values, and was more accurate in estimating MY than the Wood model. Although both models underestimated peak MY, mean, and total MY, the Wilmink model was closer to the real values.

**Conclusion:**

Models of lactation curves may have an economic impact and may be helpful in terms of herd management and decision-making as they assist in forecasting MY at any moment of lactation. Also, data obtained from modelling can help with monitoring milk performance of each cow, diet planning, as well as monitoring the health of the cow.

## INTRODUCTION

Lactation curves present milk production data throughout lactation in a graphical way. For herds equipped with an automatic milking system (AMS), such curves are constructed based on actual daily milk yield (MY) data. The AMS provides data daily while test-day milkings record milk and milking parameters once a month. The literature shows that curves constructed based on test-day data are often modelled with the use of the following functions: Wilmink, Wood, Ali-Schaeffer, Brody, Pollott, Dijkstra, Legendre polynomials [1–6]. Ordinarily, in dairy production milkings, recording systems are based on test-day records [7,8]. Since records are prepared once a month, peak yield may be overlooked. However, the AMS records milk and milking parameters daily and, therefore, a farmer is provided with a larger and more representative set of data.

Models of lactation curves may have an economic impact and may be helpful in terms of herd management and decision-making as they assist in forecasting MY at any moment of lactation. Also, data obtained from modelling can help with monitoring milk performance for each cow, diet planning, as well as monitoring the health of the cow [9,10]. After calving, MY starts to increase and peaks between the 10th and 90th day. In Poland, 80% of Polish Black and White Holstein-Friesian cows reach the lactation peak before 60 days after calving. Some authors have noted that cows with a peak day (Pday) between the 31st and 60th day of lactation have the highest MY in the 305-day lactation period [11,12]. Production- and profit-wise, the cows should reach a high MY quickly and should maintain it at that high level for a long time [13]. However, it has been pointed out that for health and production reasons, it is preferable to obtain the lactation peak in the second month after calving. In the group of primiparas, the lactation peak is usually lower than in the group of multiparas [14]. In the period of intensive milk production, at the peak of the lactation curve, a cow’s body demands energy necessary to maintain a good level of MY. A negative energy balance, which may also negatively affect animal reproduction, may occur [15].

Amongst the many functions that may be used for lactation curve analysis, Wood and Wilmink were the most frequently used. Since its development, Wood’s three-parameter function [16] has become one of the most commonly used models to evaluate lactation in dairy animals [17–19]. Karangelil et al [3], who analysed five different models used for describing the lactation curves of Chios sheep (Wood, Wilmink, Cobby and Le Du, Cappio Borlino, Djikstra), selected the Wood model as the best adjusted to daily AMS records. In 1987, Wilmink developed his function for adjusting test-day MY data [20] and since then, a number of researchers have used that model in their analysis [17,21,22]. For instance, Otwinowska-Mindur and Ptak [8], who obtained test-day MYs for Polish Holstein-Friesian cows used the Wilmink function to adjust lactation curves. Most papers describing lactation curves are based on monthly test-day data that may differ from daily records and, therefore, it is important to compare both models in order to identify the one that is better adjusted to the AMS data.

The aim of the paper was to compare the fit of data derived from daily AMS and monthly test-day records with the use of lactation curves, data was analysed separately for primiparas and multiparas.

## MATERIALS AND METHODS

The study was carried out on three Polish Black and White Holstein-Friesians (PHF) dairy herds with a total of 958 cows that calved between the years 2013 and 2015. The farms were equipped with the Lely Astronaut A4 AMS. The animals were kept in similar conditions and fed using the partial mixed ration. The animals had access to feed *ad libitum* while feed concentrates were supplied during milking. The cows were milked by the AMS and a T4C programme provided information on milking performance throughout lactation. All visits ending with a successful milking during the first two lactations were considered. Apart from the AMS records, monthly test-day records (also called test-day data) from a SYMLEK IT system (provided by the Polish Federation of Cattle Breeders and Dairy Farmers) were available. The same cows that were milked by the AMS were also recorded by the SYMLEK IT system on test days (method A4). The total dataset used in the study consisted of 395,436 milking records (364,697 derived from AMS and 30,739 from test-day records). Daily MYs were recorded in both cases.

Test-day data used in the study may represent the type of data obtained in a conventional milking system (CMS), where milkings occur during the predetermined hours and data on milk and milking parameters are recorded once a month during test-day milkings. In a CMS, the amount of data is considerably smaller, therefore, we predict that the fit of lactation curve would be better in the case of AMS derived-data. To our knowledge this is the first study that compares test-day and AMS derived data and the goodness of their fit in lactation curves, while at the same time comparing lactation curves fitted with two different mathematical models using the real daily milking records.

In order to compare both types of data and to describe the lactation curves, two best-known and widely used mathematical models were used: Wood [16] and Wilmink [20]. Statistical analysis of the AMS data and test-day data, with the use of the Gauss–Newton method, was performed separately for the 1st and 2nd lactation, and for both together [23]. The following mathematical functions were used during the analysis:

Wood function [16]:

(1) $${\text{y}}_{\text{t}}=\text{a}\times {\text{t}}^{\text{b}}\times {\text{e}}^{-\text{ct}}$$

where: y_t_, average daily MY on a particular day of lactation; t, day of lactation; a, initial MY after calving; b, parameter determining ascending slope before the peak; c, parameter determining descending slope after the peak.

Wilmink function [20]:

(2) $${\text{y}}_{\text{t}}=\text{a}+{\text{be}}^{-\text{kt}}+\text{ct}$$

where: y_t_, average daily MY on a particular day of lactation; t, day of lactation; a, initial MY after calving; b, parameter determining ascending slope before the peak; c, parameter determining descending slope after the peak; k, factor related to moment of peak yield.

Both models were compared by an analysis of variance with the use of goodness of fit measures: coefficient of determination (R^2^), mean square error (MSE), Akaike information criterion (AIC), and Bayesian information criterion (BIC). The models were fitted using the non-linear regression PROC NLIN procedure in SAS [23]. Estimates of parameters were obtained for both models (Wood, Wilmink) separately for each lactation (1st, 2nd, both lactations) and milking system (AMS, test-day data). Lower values of MSE, AIC, and BIC and a higher R^2^ indicated a better fit of the model.

Based on both models, the following information was ob tained: Pday, yield on the peak day (peak yield, PMY), daily mean yield for the whole lactation (MMY), and total yield of the lactation (TMY).

## RESULTS AND DISCUSSION

During the analysis, we found that some papers, though referring to the original papers by Wood and Wilmink [16,20], presented a slightly different Wilmink equation [3,9]. Karangelil et al [3] reported using the following equation: y = a−be^−kt^−ct (with two minus signs instead of a plus), while Macciotta et al [9] used the equation: y = a+be^kt^+ct (without a minus sign in exponentiation). In the original paper by Wilmink [20], the equation is different: y = a+be^−kt^+ct. Even with the signs in the equation changed, both Karangelil et al [3] and Macciotta et al [9] did not obtain abnormal results, which suggests that the change in Wilmink’s equation might have been only an editorial error. However, when analysing the literature available on Wilmink’s model used in fitting lactation curves, one should be at least sceptical of the equations used in these studies.

In the present study, several goodness of fit measures were calculated separately for lactations (1st, 2nd, both), for the milking system type (AMS, test-day data), and for both mathematical models (Wood, Wilmink). Table 1 presents the information criteria for each model. The results show that in all cases the Wilmink model was a better fit than Wood, as it had the lowest values of AIC, BIC, and MSE and the highest R2 values. These results stand in contradiction to the findings of some other authors. For example, the study by Elahi Torshizi et al [4], used both models to investigate lactation in Holstein cows. Based on test-day records, that study stated that the Wood model had the highest R^2^ (0.999) and the lowest root mean square error (RMSE), which suggested that it was better adjusted than the Wilmink model. Also, Elahi Torshizi et al [4] compared the different functions used for fitting lactation curves and found relatively the highest R^2^ and lowest RMSE in models Wood, Dijkstra, Rook, and Grossman. They concluded that the Wood model was close to the actual data in predicting peak yield and peak time. Also, Ferreira et al [1] showed that the Wood model compared to the Brody as well as the Dijkstra and Pollott models had the best fit (also according to AIC and R^2^ values). Karangelil et al [3] analysed lactation curves in Chios sheep milked with an AMS. Of the 5 models (Wood, Wilmink, Cobby, Cappio, Djikstra), the Wood and Wilmink models had the highest R^2^ values (0.79) while Wood also had the lowest values for AIC and BIC and the highest convergence percentage (82.1%). Silvestre et al [22] compared a total of 5 mathematical models used in modelling lactation curves based on test-day data: Wilmink, Wood, Ali and Schaeffer, Cubic Splines, Legendre Polynomials. They found that the Wilmink, Wood, and the Ali and Schaeffer models were strongly affected by the size of the sample. Melzer et al [21] suggested that with more inhomogeneous data, using the Ali and Schaeffer model may be a good solution. Other authors successfully used only the Wilmink model to describe the lactation of dairy cows [8]. The analysis of available literature suggests that both the Wilmink and Wood models are among the best models to use in modelling lactation curves; however, depending on the type of data and its homogeneity, a different model may present a better fit. Also, the source of milking data (AMS or test-day records) may affect model selection.

**Figure 1:**
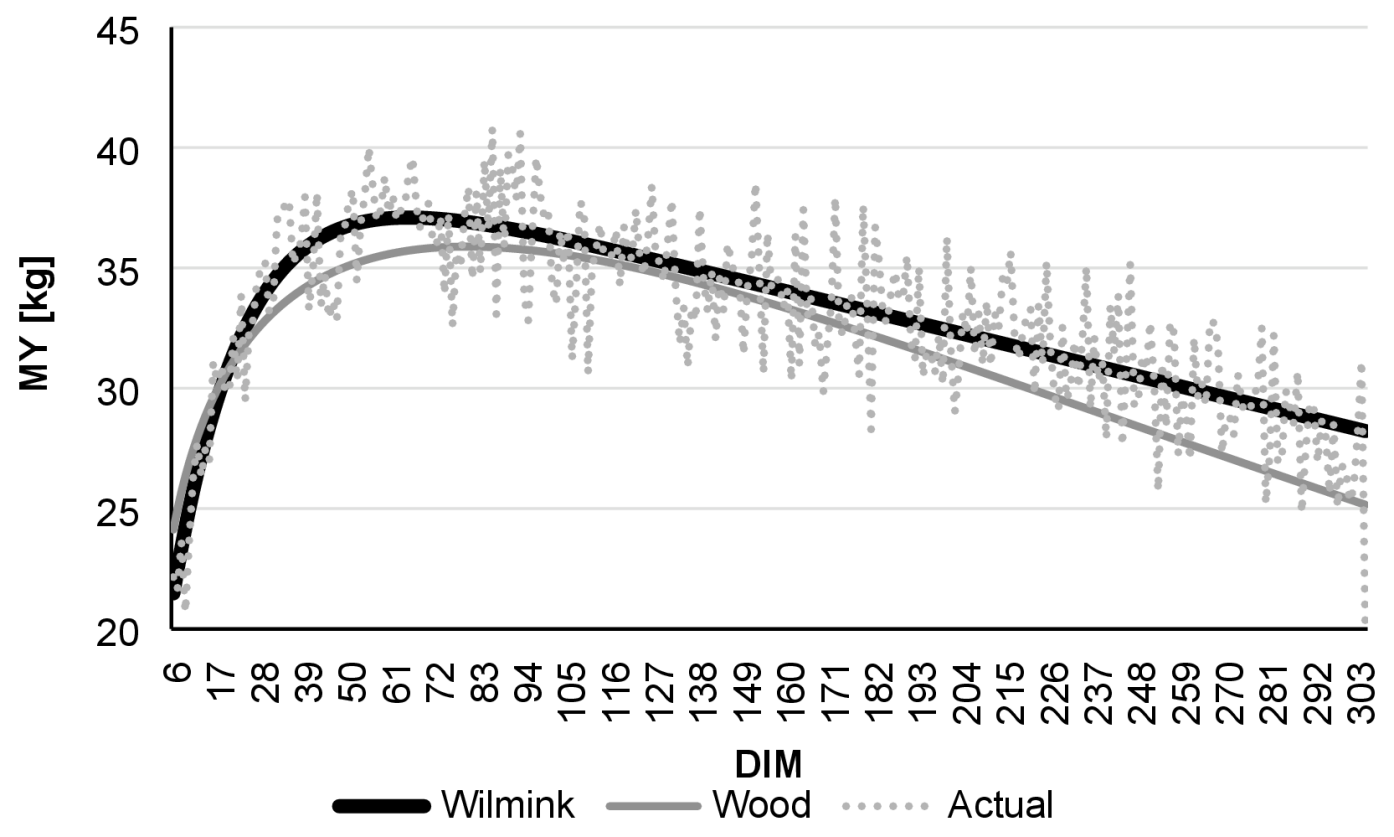
Comparison of actual daily average MY with Wilmink and Wood’s lactation curves fitted based on AMS milking data of cows in the 1st lactation. MY, milk yield; AMS, automatic milking system; DIM, days in milk.

**Figure 2:**
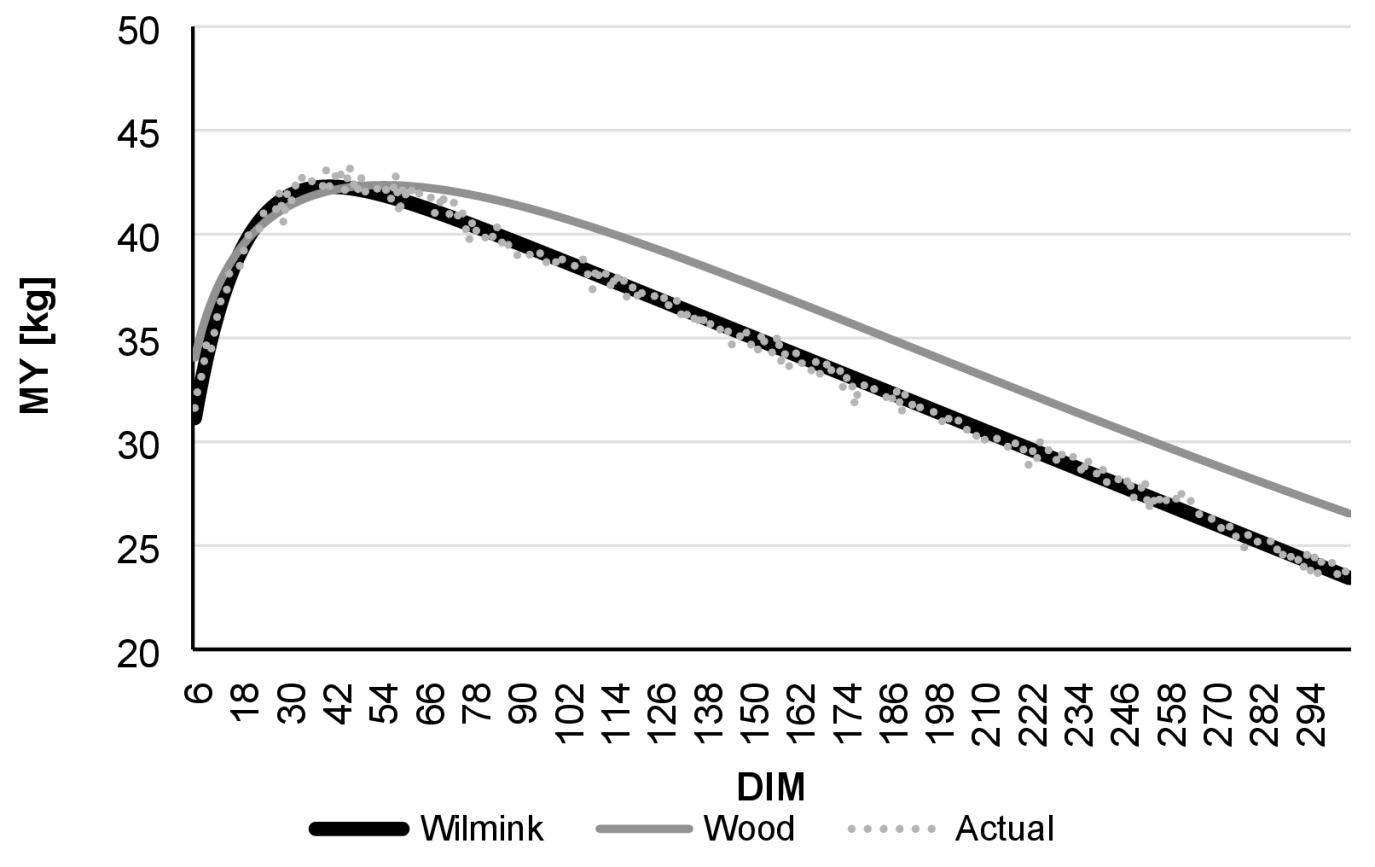
Comparison of actual daily average MY with Wilmink and Wood’s lactation curves fitted based on test-day milking data of cows in the 1st lactation. MY, milk yield; DIM, days in milk.

**Figure 3:**
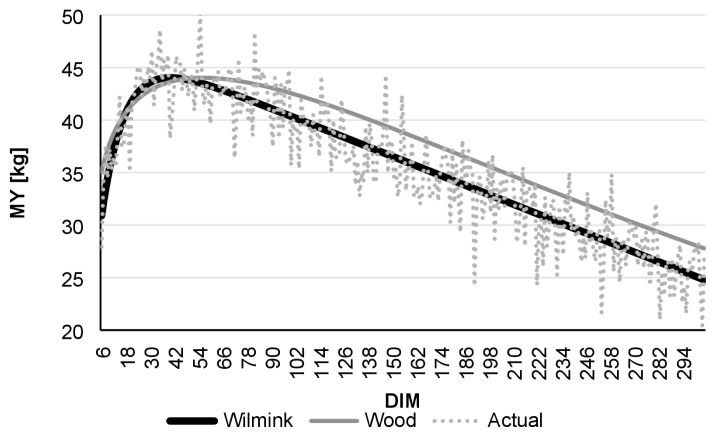
Comparison of actual daily average MY with Wilmink and Wood’s lactation curves fitted based on AMS milking data of cows in 2nd lactation. MY, milk yield; AMS, automatic milking system; DIM, days in milk.

**Figure 4:**
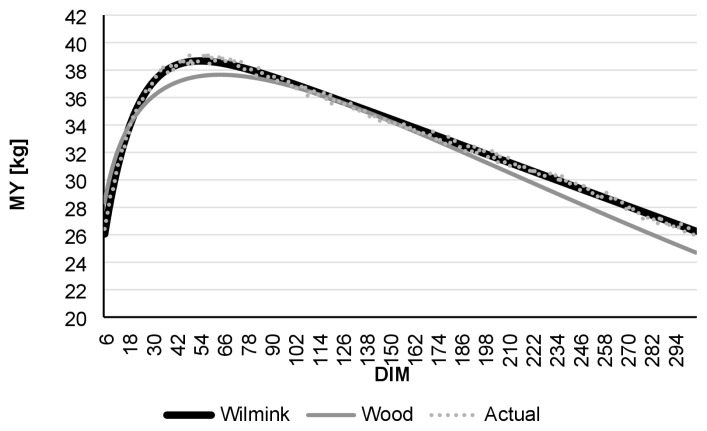
Comparison of actual daily average MY with Wilmink and Wood’s lactation curves fitted based on test-day milking data of cows in 2nd lactation. MY, milk yield; DIM, days in milk.

**Figure 5:**
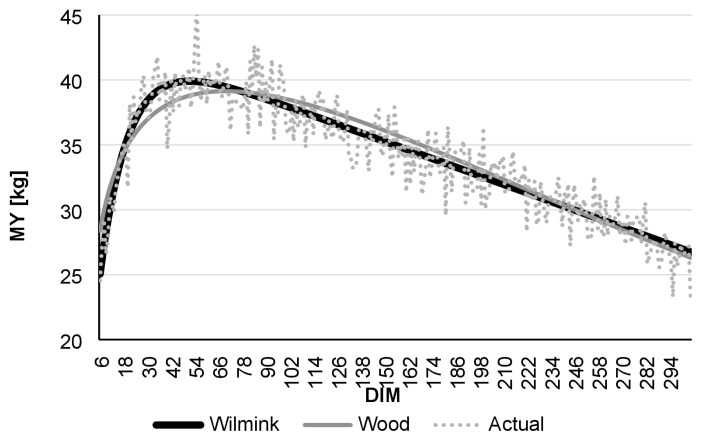
Comparison of actual daily average MY with Wilmink and Wood’s lactation curves fitted based on AMS milking data of cows in 1st and 2nd lactation together. MY, milk yield; AMS, automatic milking system; DIM, days in milk.

**Figure 6:**
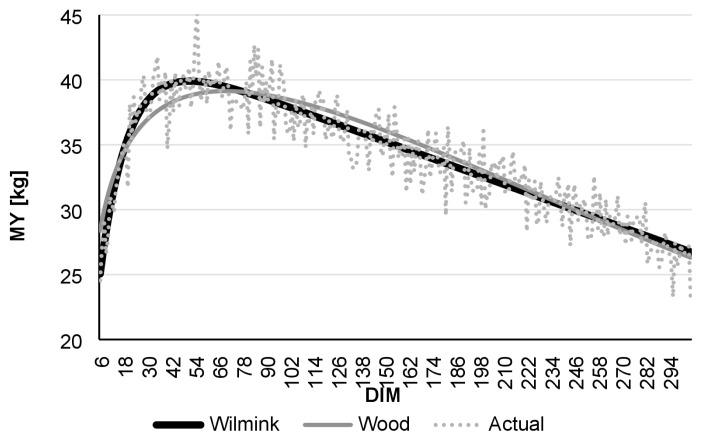
Comparison of actual daily average MY with Wilmink and Wood’s lactation curves fitted based on test-day milking data of cows in 1st and 2nd lactation together. MY, milk yield; DIM, days in milk.

Comparing daily AMS and monthly test-day data, R ^2^ was found to be highest in the case of daily data. Even though the Wood model had lower R^2^ values than Wilmink, it still showed better adjustment with data derived from the AMS than to real-data (Table 1). Figure 1 to 6 present lactation curves based on the Wood and Wilmink models constructed separately for AMS and test-day data for each lactation number. The best fit was observed with the AMS data and for the curve constructed based on the Wilmink function. All functions fitted with the use of the Wood and Wilmink models had a standard shape with a visible period of growth, a peak point, and a decrease. The results show that curves created from daily records provided by an AMS are better adjusted to the actual MY data, which is in agreement with the findings of other authors. It has been pointed out that atypical lactation curves are less likely to occur in an AMS than in a traditional one (CMS), also the peak day is less likely to be missed [8,9].

Table 1 also presents curve parameters calculated for all groups. Both the Wood and Wilmink models classify lactation curves as standard or atypical based on the *b* and *c* parameters. In the case of the Wood model, if the *b* and *c* parameters are above zero, the curve is a standard one, while in the Wilmink model the standard curve has negative values for *b* and *c* [24]. In the present study, regardless of the lactation number or milking system, both parameters were positive in the Wood model and negative in the Wilmink one, proving lactation curves to be standard.

The present study divided data into several clusters based on lactation number, milking system, and model used to fit the curve. Compared to the daily data gathered throughout the whole lactation, it was noted that the Wilmink model constructed on AMS data was better adjusted. Regardless of the lactation number, it estimated Pday and PMY, as well as mean and total MY, better than the Wood model (Table 2). During the first lactation, the Pday estimated by the Wilmink model fell on the 68th day, with Wood on the 76th, while analysis of the real data showed the highest MY on the 71st day of lactation. During the second lactation, the Wilmink model underestimated Pday by 5 days (41st day), while the Wood model overestimated it by 9 days (55th day). Analysis of the whole dataset, without distinguishing lactation number, showed that the Wilmink predicted Pday to be 4 days earlier while Wood was 6 days later than in reality. Although both models underestimated PMY, mean and total MY, the Wilmink model was closer to the real values. Analysis of test-day records showed bigger differences. While in the case of the 1st and 2nd lactation the Wood model estimated Pday more closely to the real peak day, the Wilmink function fit the MY better. It is worth mentioning that in the case of test-day records, it is easy to miss the actual peak day [9]. Other authors who analysed MY data usually did so based on test-day records. Many of them pointed out Wood to be the best fit to the lactation curve [10,19,25]. Janković et al [26], who used the Wood model to estimate both Pday and PMY, reported that the Wood model miscalculated the Pday (estimated Pday was 61.1st day while the actual data showed the peak on the 55th day) and underestimated the maximum MY (estimated 54.2 kg versus actual 55.46 kg). In the study by De Marchi et al [27], the highest MY was noted near the 50th day of lactation, during which time significant differences between MY levels were observed between the test-day and AMS data, with AMS data showing higher average MY. Løvendahl and Chagunda [28] found the highest MY was observed between the 42nd and 57th day of lactation. Rowlands et al [6] compared peak days for primiparas and multiparas, reporting that primiparas reached lactation peak in the 10th week and multiparas in the 7th. Ferreira et al [1], who used different models to fit lactation curves for Holstein cows, reported that the Wood model (for the 75% quartile) estimated the peak day to be on the 68th day in the first lactation and 46th in the second. Similar to the results from the present study, the MY on the peak day was higher during the second lactation (28.18 kg in the 1st and 34.11 kg in the 2nd lactation). Khalifa et al [18] compared peak day and yield depending on calving season, and found that Pday varied (84.86th day of lactation if calving occurred in the autumn or winter season, 82.49th day in spring, and 87.73rd in summer) while PMY was similar (around 23 kg).

During the first lactation, cows milked by AMS gave an average of 32.7 kg of milk per day, during the 2nd, 34 kg/d (Table 2). During the whole period of study, a cow milked in the robot produced an average of 9,970.65 kg of milk per lactation, with more milk being produced during the second lactation (10,201.80 kg) than in the first (9,796.70 kg). Based on data collected daily by AMS and monthly by the SYMLEK IT system (test-day data), the Pday and PMY was calculated. Test-day milkings occurred once a month, with cows being in different day in milk, which contributed to the difference in the peak day and peak MY calculated from both data sources. Pday and PMY in the AMS were the 58th day and 39.34 kg, respectively, while test-day data resulted in the 55th day and 45.74 kg, respectively. Cows milked by the AMS reached their peak yield later during the first lactation (71st day) and had lower PMY (37.12 kg) than cows in their second lactation (46th day and 43.3 kg). Data from test-day milkings show Pday to be on the 86th day in the 1st lactation and 55th in the second. While in the present study the MY for all cows milked in the AMS was 39.34 kg, the value was higher than the one reported by Ettema and Santos [29]. They found that throughout lactation cows produced an average of 33.4 kg of milk per day; however, between the 50th and 200th day of lactation, the cows produced more than 35 kg of milk per day. Elahi Torshizi et al [4], who based their analysis on test-day data, reported that Holstein cows produced overall 29.8 kg of milk per day. However, they also noticed a difference in milk performance between different herds and suggested that it was caused by variation in management, feeding, as well as by some environmental factors.

## CONCLUSION

Various authors suggest that different models may be useful in analysing data from AMS and from test-day milkings. The Wood model is known to overestimate MY before the peak [9], the model also has a big margin of error for estimating total lactation MY [3]. A mathematical model’s usefulness depends greatly on its ability to reflect the milk production process. Most studies described in the literature are based on test-day data; therefore, we aimed to compare models based on both test-day and AMS data to determine which mathematical model (Wood, Wilmink) would be the better fit. It has been shown that models based on AMS records were a better fit and generated less atypical lactation curves. Models based on test-day records contain less data and a peak may be easily missed [8]. The results of the present study show that lactation curves constructed from data derived from the AMS indeed were a better fit regardless of the model used. Moreover, they suggest that the Wilmink model may be a better fit for modelling the lactation curve of PHF cows milked by an AMS. While for test-day data the Wood model predicted Pday better than the Wilmink model, the Wilmink model was more accurate in estimating MY.

## Figures and Tables

**Table 1:** Comparison of goodness of fit measures and lactation curve parameters between mathematical models (Wood, Wilmink), milking system types (AMS, test-day data) and lactation numbers (1st, 2nd, and both)

Lactation number	Milking system type	Model	MSE	AIC	BIC	R2	a	b	c	k
1st lactation	AMS	Wood	0.644	−516.397	−514.383	0.951	16.461	0.229	0.003	-
		Wilmink	0.304	−700.400	−698.373	0.990	40.227	−23.579	−0.041	0.049
	Test-day data	Wood	1.729	326.400	328.430	0.681	16.007	0.239	0.003	-
		Wilmink	1.674	307.320	309.349	0.719	40.421	−26.013	−0.040	0.055
2nd lactation	AMS	Wood	0.678	−226.626	−224.590	0.985	25.792	0.165	0.003	-
		Wilmink	0.410	−523.528	−521.500	0.994	46.322	−23.516	−0.075	0.078
	Test-day data	Wood	2.370	499.030	501.057	0.830	26.379	0.169	0.003	-
		Wilmink	2.280	476.996	478.995	0.843	47.966	−27.842	−0.076	0.086
Both lactations	AMS	Wood	0.631	−269.600	−267.579	0.972	20.447	0.193	0.003	-
		Wilmink	0.242	−832.690	−830.660	0.995	42.422	−23.008	−0.053	0.060
	Test-day data	Wood	1.634	292.840	294.870	0.834	19.867	0.209	0.003	-
		Wilmink	1.489	237.760	239.788	0.867	43.519	−27.433	−0.055	0.069

MSE, mean square error; AIC, Akaike information criterion; BIC, Bayesian information criterion; R^2^, coefficient of determination; AMS, automatic milking system.

**Table 2:** Comparison of lactation traits between mathematical models (Wood, Wilmink), milking system types (AMS, test-day data) and lactation numbers (1st, 2nd, and both)

Lactation number	Milking system type	Model	Pday (d)	PMY (kg)	MMY (kg)	TMY (kg)
1st lactation	AMS	Real data	71	37.199	32.656	9,796.70
		Wood	76	35.330	31.154	9,346.19
		Wilmink	68	36.597	32.627	9,787.96
	Test-day data	Real data	86	40.789	32.940	9,882.10
		Wood	80	35.885	31.772	9,531.52
		Wilmink	65	37.092	33.036	9,910.84
2nd lactation	AMS	Real data	46	43.303	34.006	10,201.80
		Wood	55	42.363	36.051	10,815.20
		Wilmink	41	42.287	34.005	10,201.58
	Test-day data	Real data	55	50.163	35.454	10,636.14
		Wood	56	44.029	37.573	11,271.95
		Wilmink	40	44.033	35.476	10,642.73
Both lactations	AMS	Real data	58	39.340	33.235	9,970.65
		Wood	64	37.656	32.621	9,786.31
		Wilmink	54	38.659	33.262	9,978.51
	Test-day data	Real data	55	45.740	34.025	10,207.56
		Wood	70	39.134	34.192	10,257.66
		Wilmink	51	39.901	34.060	10,217.98

Pday, peak day (days); PMY, yield at the peak day (kg); MMY, daily mean yield for the whole lactation (kg); TMY, total yield of a lactation (kg); AMS, automatic milking system.
